# HIV-1 coreceptor tropism: A syllogistic connection with The Veterans Aging Cohort Study Index and the CD4/CD8 ratio

**DOI:** 10.1371/journal.pone.0212882

**Published:** 2019-02-28

**Authors:** Armando Leone, Nicolò de Gennaro, Claudia Fabrizio, Luigia Scudeller, Luciana Lepore, Antonella Lagioia, Grazia Punzi, Annalisa Saracino, Gioacchino Angarano, Laura Monno

**Affiliations:** 1 Department of Biomedical Sciences and Human Oncology, Clinic of Infectious Diseases, University of Bari, Italy; 2 Scientific Direction, Clinical Epidemiology Unit, Fondazione Policlinico IRCCS San Matteo, Pavia, Italy; University of Alabama at Birmingham, UNITED STATES

## Abstract

**Background:**

The association between X4 virus and an increased risk of non-AIDS-events has been reported. Morbidity/mortality due to non-AIDS events, which are properly predicted by the CD4/CD8 ratio and VACS index, have become particularly remarkable in HIV-infected patients receiving effective combined antiretroviral therapy (cART).

**Methods:**

We verified the validity of the syllogism: as HIV-tropism (CRT) contributes to the onset of non-AIDS events which are successfully predicted by the CD4/CD8 ratio and VACS index, then CRT correlates with these two variables. The CD4/CD8 ratio and VACS index at baseline and overtime were analyzed according to CRT tested before the first successful cART regimen in newly-diagnosed patients.

**Results:**

Patients with R5 variants had a significantly lower baseline VACS percentage risk [mean (95%CI):18.2%(16.1–20.3) *vs* 24.3%(18.2–22.5), p = 0.002] and higher baseline CD4/CD8 ratio [mean (95%CI):0.43 (0.38–0.47) *vs* 0.28 (0.19–0.36), p = 0.002] than non-R5 patients. After an initial drop, VACS increased again in R5 and non-R5 patients and the two trend curves almost overlapped. The CD4/CD8 ratio had an increasing trend in both R5 and non-R5 patients; however, even though non-R5 patients had a greater gain of CD4+, they maintained a lower CD4/CD8 ratio at any time point.

**Conclusion:**

Our study confirms an association between pre-therapy CRT, CD4/CD8 ratio and VACS. A successful cART regimen positively affects the CD4/CD8 ratio; however, the disadvantage conferred by a non-R5 CRT is maintained overtime. The restoration of VACS in all patients could be directly due to variables included in the VACS calculation and to factors that adversely influence these variables.

## Introduction

Combination antiretroviral therapy (cART) has transformed HIV infection from an incurable disease into a long term chronic condition; however, although the evolution of HIV infection to full-blown AIDS is now preventable, non-AIDS events are increasingly contributing to morbidity and mortality in HIV-infected patients on cART [1–3]. As in the general population, multiple factors facilitate the occurrence of morbid events either in naive and treated HIV-infected patients even with high CD4 cell counts (≥350 cells/mm^3^); nevertheless, the higher incidence of these events in HIV-infected patients compared to their HIV-negative counterparts suggests the existence of additional and specific factors.

A number of studies assessed the relationship between co-receptor tropism (CRT) and HIV disease progression [4–7]; in particular, patients with a dual/mixed (DM) or X4 strain have a greater and a more rapid CD4 cell decline and progression to AIDS compared to persons with a R5 virus [5]. More recently, Maffongelli et al [8] demonstrated that patients harbouring X4 strains also have a higher propensity to develop non-AIDS events during their first ART regimen, thereby establishing an association between CXCR4 coreceptor usage and non-AIDS comorbidities.

The CD4/CD8 ratio has become an impressive marker for immune dysfunction among HIV-infected patients [9] and, unlike the CD4 cell count, it can be used by clinicians to identify patients at risk of non-AIDS-related events [10,11].

The Veterans Aging Cohort Study Index (VACS Index) has been proposed as a tool for predicting the risk of all-cause mortality among HIV-infected persons [12]; in fact, the VACS index, that combines HIV and non-HIV biomarkers (hemoglobin, aspartate aminotransferase, alanine aminotransferase, platelet count, creatinine levels and hepatitis C virus serostatus), reflects the multisystem injury among people living with HIV and it has been validated as an instrument to recognize and check signs of non-AIDS comorbidities [12–14].

Given the above, we hypothesized that the following syllogism should be appropriate: (major premise) if CRT is associated with non-AIDS events and (minor premise) the risk of non-AIDS events is properly predicted by the CD4/CD8 ratio and the VACS index, then (conclusion) CRT does associate with these two latter parameters and influence their trend. Therefore, in this study we aimed to verify whether HIV coreceptor tropism assessed before the first successful cART regimen correlates with baseline CD4/CD8 ratio and VACS index, and to determine whether it is associated with changes in CD4/CD8 ratio and VACS index overtime.

## Patients and methods

Patients were eligible for the study if they: (i) were newly diagnosed HIV-positive patients, (ii) naïve to previous antiretroviral therapy; (iii) initiated their first successful cART; (iiii) had co-receptor tropism coincident with HIV diagnosis and, in any case, before starting cART. Eligibility criteria also included the availability of demographic, laboratory and clinical data.

For patients selected, laboratory indicators of HIV infection (CD4+ and CD8+ counts; HIV-RNA) and of organ system injury, including hemoglobin, platelets, aspartate and alanine transaminase (AST and ALT), creatinine, and hepatitis C virus (HCV) infection status, at baseline (time of ART initiation) and, when applicable, at 6, 12 and 24 months after ART initiation were recorded into a dedicated database. Pol sequences of patients at the time of their first HIV-positive testing were used for the assignment of HIV subtype and the estimation of the duration of HIV infection.

For all patients the CD4:CD8 ratio and the VACS index were calculated at baseline and all subsequent visits.

The calculation of VACS Index generates a score by summing pre-assigned points for age, sex, race, CD4 count, HIV-1 RNA, hemoglobin, AST, ALT, platelets, the Fibrosis-4 score (FIB-4) [15], creatinine, renal glomerular filtration rate (eGFR) and HCV status (https://vacs.med.yale.edu/calculator/IC). The score is weighted to indicate increasing risk of all-cause 5-year mortality with increasing score and converted into a percentage risk.

### Duration of infection and HIV-1 subtyping

The duration of HIV infection was estimated by calculating the proportion of ambiguous nucleotides of pol (protease, PR and reverse transcriptase, RT) sequences on the first available plasma sample after HIV diagnosis: ≤0.2% ambiguity signified a recent infection (≤1yr), vs older infections [16]. In fact, although most individuals are infected with a single HIV founder virus variant [17], a linear increase in viral diversity of 0.2% ambiguous bases per year has been described during the first eight years of infection [18].

REGA HIV-subtyping Tool 3.0 (https://hivdb.stanford.edu) was used for the assignment of HIV subtype [19]; for the purpose of this study, patients were classified as infected with either B or non-B HIV-1 variants.

### V3 sequence analysis for viral tropism

CRT was determined by gp120 sequencing [20] on plasma samples and inferred with the geno2pheno coreceptor algorithm (http://coreceptor.bioinf.mpi-inf.mpg.de/) with a false positive rate (FPR) of 10% according to current guidelines [21].

Patients were classified as infected with a R5 strain (FPR>10%) or a non-R5 variant (FPR ≤10%) (either X4 and dual tropic strain).

### Statistical analysis

Descriptive statistics were produced for demographic, clinical and laboratory characteristics of patients. Mean and standard deviation (SD) are presented for normally distributed variables, and median and interquartile range (IQR) for non-normally distributed variables, number and percentages for categorical variables. Groups (R5 vs non-R5) were compared with Pearson’s χ2 test (Fisher exact test where appropriate) for categorical variables and with parametric or nonparametric tests, according to data distribution, for continuous variables; Shapiro Wilk's and Kolmogorov-Smirnov test, as well as visual methods, were applied to test for normality. Log-transformed variables were used in models in case of deviation from normality.

To assess the factors mediating or explaining the association between CRT and VACS percentage risk, a multivariable logistic regression model of tropism (non-R5 vs R5) with all variables included in the VACS calculation was fitted.

To assess the VACS percentage risk and the CD4/CD8 ratio overtime univariate and multivariate multilevel generalized linear models were applied, with patient’s ID as random effect, time as fixed effect; interaction between CRT and time was included to assess difference between non-R5 and R5 patients in parameter trend overtime. VACS percentage risk was log-transformed to achieve normalization prior to input in models.

In multivariate models, after excluding variables included in VACS calculation or those collinear to them, variables (besides CRT and time) associated to VACS percentage risk at univariate analysis were included, with no further selection (the final model therefore included tropism and its interaction with time, subtype, gender, HIV risk group). The same strategy was applied for building models for CD4/CD8 ratio.

Fit of the linear models was assessed by means of the R-squared. In all cases, 2-tailed tests were used. P-value significance cut-off was 0.05. Stata computer software version 14.0 (Stata Corporation, 4905 Lakeway Drive, College Station, Texas 77845, USA) was used for statistical analysis.

### Ethics

The study did not require approval from the ethics committee, according to the Italian law, since it was performed in the context of normal clinical routines (art.1, leg. decree 211/2003). However, all patients referring to our institute provided consent for the use of their data for research purposes. In any case, data were previously anonymized, according to the requirements set by Italian Data protection Code (leg. Decree 196/2003).

## Results

### Patients’ characteristics

Four hundred and fifty-four subjects were newly diagnosed with HIV infection between 2007–2016 at our institute. For 417 (91.8%) of these subjects, coreceptor tropism was successfully assessed at the time of HIV diagnosis (20.8% non-R5 and 79.1% R5), and 328/417 patients who initiated their first successful cART regimen were included in the study. On the whole, 67 (20.4%) and 261 (79.5%) subjects harboured a non-R5 and a R5 strain, respectively. In Table 1 the baseline characteristics of the study population are reported.

**Table 1:** Baseline characteristics of patients according to the coreceptor tropism of HIV-1.

		Non-R5 patients (n = 67)	R5 patients (n = 261)	p-value
Gender	Males	54 (80.6)	216 (82.7)	0,05
Age in years	mean (SD)	39.5 (±12.5)	37.8 (±11.8)	0.25
Risk factor for HIV infection	Heterosexual	26 (38.8)	112 (42.9)	0.45
	MSM	27 (40.3)	113 (43.3)	
	IDU	3 (4.5)	6 (2.3)	
	Other/unknown	11 (16.4)	30 (11.5)	
Nationality	Italians	58(86.6)	227(87.0)	0.05
HIV subtype	B	55(82.1)	165(63.2)	**0.003**
	non-B	12 (17.9)	96 (36.8)	
Year of diagnosis	median (IQR)	2011 (2008–2013)	2011 (2009–2013)	0.22
Estimated duration of HIV infection	< 1 year	25 (37.3)	103 (39.5)	0.43
	> 1 year	42 (62.7)	158 (60.5)	
AIDS diagnosis	yes	18 (26.9)	40 (15.3)	**0.002**
Viral load at diagnosis (log10 copies/ml)	median (IQR)	4.85 (4.23–5.55)	4.77 (4.14–4.32)	0.15
*Category of HIV-RNA (copies/ml)	<500	1 (25.0)	3 (75.0)	0.59
	500 to 99,999	36 (18.5)	158 (81.4)	
	>100,000	30 (23.0)	100 (77.0)	
CD4+ cell count at diagnosis (cells/mm3)	median (IQR)	168 (48–372)	327 (151–484)	**0.0005**
*Category of CD4+ cell count (cells/mm3)	≥ 500	9 (13.0)	60 (87.0)	**0.0072**
	350 to 499	11 (14.8)	63 (85.2)	
	200 to 349	9 (14.0)	55 (86.0)	
	100 to 199	13 (26.0)	37 (74.0 =	
	50 to 99	7 (36.8)	12 (63.2)	
	< 50	18 (34.6)	34 (65.3)	
*Category of Hemoglobin (g/dl)	> 14	28 (19.1)	118 (80.8)	0.16
	12 to 13.9	19 (16.6)	95 (83.3)	
	10 to 11.9	13 (27.0)	35 (72.9)	
	< 10	7 (35.0)	13 (65.0)	
*Category of FIB-4 Index	< 1.45	55 (20.1)	218 (79.8)	0.056
	1.45 to 3.25	10 (23.8)	32 (76.2)	
	> 3.25	3 (23.0)	10 (76.9)	
* Category of eGFR (mL/min)	> 60	65 (20.4)	253 (79.6)	0.066
	45 to 59.9	1 (20.0)	4 (80.0)	
	30 to 44.9	0	1	
	< 30	1 (25.0)	3 (75.0)	
*Hepatitis C coinfection	Yes	4 (28.5)	10 (71.5)	0.43

* components of the VACS Index

NB: figures indicate number of patients (percentage) unless otherwise specified

MSM = men who have sex with men; IDU = intravenous drug users; AIDS = acquired immunodeficiency syndrome; FIB-4 Index = Fibrosis-4 Index for Liver Fibrosis; eGFR = Estimated Glomerular Filtration Rate

Patients infected with a non-R5 virus had a CD4 cell count at diagnosis significantly lower than patients with a R5 variant [median (IQR): 168 (48–372) vs 327 (151–484), p = .0005] and were more likely to be diagnosed as AIDS patients (26.9% vs 15.3%, p = .002). No association was observed between CRT and sex, age, year of diagnosis, risk factor for the acquisition of infection, viral load at diagnosis, and estimated duration of HIV infection. However, non-R5 strains were significantly more frequent among patients infected with subtype B HIV-1 than in subjects with non-B subtype (82.1% vs 17.9%; p = .003). In particular, this difference applied to patients with an older (>1 year) infection (non-R5 prevalence: B 85.71% vs non-B 14.29% p = .004), but not to patients with recent infection (non-R5 prevalence: 76% vs 24% p = .142).

Among components of the VACS index, the only statistically significant difference between non-R5 and R5 patients was the proportion of patients with low CD4 cell count (p = .0072) (Table 1).

Overall, the median time from HIV diagnosis to ART initiation was 2.99 (IQR, 1.38–12.09) without a significant difference between non-R5 and R5 patients (2.51 [IQR, 1.11–6.31] months vs 3.28 [IQR, 1.46–12.9] months; p = 0.10). Within the first six months of cART, the rate of viral suppression was 98.2% for non-R5 patients and 97.4% for R5 (p = 0.69).

### Coreceptor tropism and VACS index

**Fig 1:**
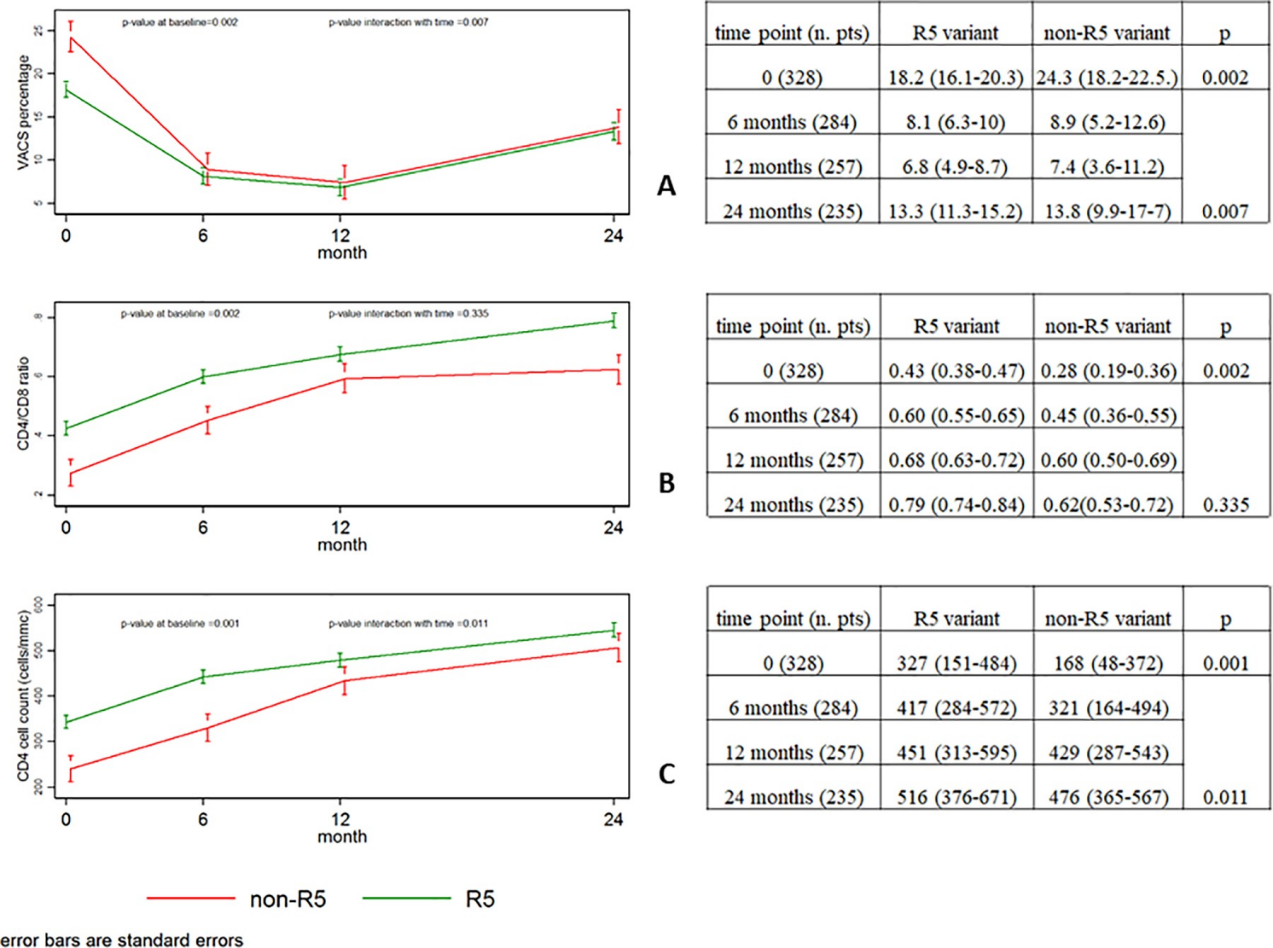
**Coreceptor tropism and VACS percentage risk (A), CD4/CD8 ratio (B) and CD4 cell count (C): association at diagnosis and changes over time**.

Patients with R5 variants had a significantly lower baseline VACS percentage risk than subjects with non-R5 strains [mean (95% CI): 18.2% (16.1–20.3) *vs* 24.3% (18.2–22.5), p = 0.002] (Fig 1A). In R5 patients the VACS percentage significantly dropped at 6 and 12 months [mean (95% CI): 8.1% (6.5–10) and 6.8% (4.9–8.7)], and increased again at 24 months to 13.3% (11.3–15.2) (all p<0.001). In patients infected with a non-R5 strain, the VACS index showed an even stronger decline at 6 months; thereafter, the initial gap between R5 and non-R5 patients greatly narrowed over time (p-value for interaction with time = 0.007).

Among all variables included in the VACS calculation, only the CD4 cell count was independently associated with CRT (Table 2).

**Table 2:** Association between coreceptor tropism and individual VACS components.

VACS PARAMETERS	ODD RATIO (95% IC) for harbouring a non-R5 virus	p-value
Age	1.01 (0.98–1.04)	0.36
CD4^+^ cell/mm^3^	0.82 (0.70–0.96)	**0.016**
Viral load (log_10_ copies/mL)	1.05 (0.74–1.48)	0.79
Hemoglobin	1.00 (0.85–1.17)	0.98
FIB-4	0.99 (0.96–1.02)	0.47
eGFR	1.01 (1.00–1.03)	0.12
HCV serostatus	1.56 (0.44–5.46)	0.49

Multivariable logistic regression model. FIB-4 = Fibrosis-4 Index for Liver Fibrosis; eGFR = estimated glomerular filtration rate.

At multivariable analysis, non-R5 patients had a higher VACS percentage risk than R5 patients, after adjusting for sex, HIV risk factor and subtype (Table 3).

**Table 3:** Association between patients’ and viral characteristics and VACS index percentage risk.

		Univariate			Multivariable *		
Variable	Category	Folds difference	(95% CI)	p-value	Folds difference	(95% CI)	p-value
Coreceptor tropism	R5	Reference	Reference				
	Non-R5	1.30	(1.00–1.69)	0.04	1.43	(1.11–1.85)	0.006
Gender	Male	Reference	Reference				
	Female	2.53	(2.03–3.15)	<0.001	1.59	(1.2–2.1)	0.001
Nazionality	Italians	Reference	Reference				
	Non Italians	2.28	(1.65–3.16)	< 0.001			
HIV subtype	Non-B	Reference	Reference				
	B	0.72	(0.56–0.93)	0.014	0.81	(0.64–1.03)	0.09
Age	Per each year	1.06	(1.03–1.05)	< 0.001			
CD4+ cell/μl at diagnosis	Per each 100 cell/μl	0.79	(0.76–0.82)	< 0.001			
Viral load (log_10_ ml) at diagnosis	Per each Log_10_	1.18	(1.06–1.33)	< 0.003			
Risk factor	heterosexual	Reference	Reference				
	MSM	0.4	(0.319–0.5)	< 0.001	0.49	(0.37–0.64)	< 0.001
	IDU	0.88	(0.54–1.44)	0.62	1.1	(0.65–1.87)	0.716
	other/Unknown	0.49	(0.34–0.71)	< 0.001	0.59	(0.40–0.86)	0.006

* Multivariable model also include time and its interaction with tropism (for details, see text); MSM = men who have sex with men; IDU = intravenous drug users

### Coreceptor tropism and CD4/CD8 ratio

Patients with R5 strains had a significantly higher baseline CD4/CD8 ratio than non-R5 patients [mean (95%CI): 0.43 (0.38–0.47) *vs* 0.28 (0.19–0.36), p = 0.002]. After treatment initiation, the CD4/CD8 ratio significantly increased over time in patients with R5 variants; at 6, 12 and 24 months the mean CD4/CD8 ratio was 0.60 (95%CI) (0.55–0.65), 0.68 (0.63–0.72); and 0.79 (0.74–0.84), respectively (all p<0.001). In non-R5 patients, the CD4/CD8 ratio increase was similar (p-value for interaction with time = 0.335), however at any time-point non-R5 patients maintained a lower CD4/CD8 ratio than R5 patients (Fig 1B).

At multivariable analysis, patients harbouring non-R5 variants had a lower CD4/CD8 ratio than R5 patients, after adjusting for sex, HIV risk factor and subtype (Table 4).

**Table 4:** Association between patients’ and viral characteristics and the CD4/CD8 ratio.

		Univariate			Multivariable *		
Variable	Category	Folds difference	(95% CI)	p-value	Folds difference	(95% CI)	p-value
Coreceptor tropism	R5	Reference	Reference				
	Non-R5	-0.142	(-0.218/-0.65)	< 0.001	-0.17	(-0.25/-0.10)	< 0.001
Gender	Male	Reference	Reference				
	Female	0.068	(-0.04/0.17)	0.21	0.17	(0.06–0.29)	0.004
Nazionality	Italians	Reference	Reference				
	Non Italians	-0.108	(-0.21/0.003)				
HIV subtype	Non-B	Reference	reference				
	B	0.046	(-0.02/0.12)	0.22	0.08	(0.00/0.15)	0.045
Age	Per each year	-0.006	(-0.009/0.003)	< 0.001			
CD4+ cell/μl	Per each 100 cell/μl	0.08	(0.072/0.095)	< 0.001			
at diagnosis							
Viral load (log_10_ ml) at diagnosis	Per each log_10_	-0.11	(-0.15/0.079)	< 0.001			
Risk factor	heterosexual	Reference	Reference				
	MSM	-0.063	(-0.01/0.14)	0.11	0.13	(0.05/0.21)	0.002
	IDU	0.06	(0.19/0.05)	0.27	-0.01	(-0.17/0.16)	0.95
	other/						
	Unknown	0.14	(0.01/0.27)	0.02	0.20	(0.07/0.32)	0.003

*Multivariable model also include time and its interaction with tropism (for details, see text); MSM = men who have sex with men; IDU = intravenous drug users

### Coreceptor tropism and CD4

To verify whether the expected immune recovery could have influenced the CD4/CD8 ratio, the trend overtime of the CD4 cell count after ART initiation was also evaluated (Fig 1C). CD4+ cells count increased among patients infected with a R5 strain at 6, 12 and 24 months. Baseline CD4 cell count in non-R5 patients was significantly lower with respect to R5 patients (p = 0.001), thereafter, non-R5 patients had a greater increase of CD4+ than their R5 counterparts with a progressive significant reduction of the initial difference (p-value for interaction with time = 0.011).

## Discussion

While fewer and fewer AIDS-related manifestations are being registered among HIV positive subjects, the proportion of non-AIDS events is increasing, so that there is still an excess morbidity/mortality risk for HIV infected persons compared with the general population. Admittedly, the pathogenesis of non-AIDS events is multifactorial and includes, among other factors, the immune activation that characterizes the HIV infection [22]. Although CRT on HIV provirus does not seem to influence the persistent immune activation and inflammation in HIV-infected patients [23], to our knowledge, only limited data are available that directly correlate pre-therapy CRT of replicating HIV with the onset of non-AIDS events during treatment.

Currently, the risk of non-AIDS events appears to be properly identified by using some surrogate markers such as the CD4/CD8 ratio and the VACS index [24,25]. Subjects with even an optimal CD4 T cell recovery but a persistent elevation and expansion of CD8 T cells still have an increased immune activation and a higher risk of non-AIDS morbidity and mortality [26]. The utility of the VACS index as a prognostic index in HIV infection is also almost obvious: as the VACS index combines “HIV” and “non-HIV” markers it provides an improved risk estimation of all cause morbidity and mortality [12].

Herein, we attempted to verify a possible association between pre-therapy HIV-coreceptor usage, the CD4/CD8 ratio and the VACS index at baseline and overtime to ascertain the validity of the syllogism: HIV-tropism contributes to the onset of non-AIDS events; non-AIDS events are successful predicted by the CD4/CD8 ratio and VACS index; CRT correlates with the CD4/CD8 ratio and VACS index.

The association between an X4 virus and an increased risk of non-AIDS-events development has been optimally described by Maffongelli and coworkers [8] in a cohort of patients receiving an effective ART regimen. The finding was even more convincing in consideration of the significant correlation that the authors also found between a decreasing false positive rate and the emergence and number of non-AIDS events. The most likely explanation for this association might rely on the increased generalized immune activation that has been attributed to X4 strains [27]. In fact, the infection and depletion of central memory CD4+ T cells (CD4+ TCM) (that are CCR5 negative and, in the absence of other stimuli, can only be infected by X4 viruses because they do express CXCR4) is hypothesized to contribute to the establishment of chronic immune activation [28]. Accordingly, we found that HIV-1 CRT in newly diagnosed patients before the initiation of their first successful ART regimen was associated with both the CD4/CD8 ratio and VACS index. In particular, non-R5 patients had a significantly lower baseline CD4/CD8 ratio and a higher VACS percentage risk than their R5 counterparts. This result does not contradict previous observations [23], in fact, it is plausible that the association between CXCR4 tropism and inflammation should require the active viral replication [29].

During the two years following cART initiation, non-R5 patients experienced a greater increase of CD4 cell count than R5 patients, nevertheless non-R5 patients appeared to maintain a lower CD4/CD8 ratio which signifies a persistent immune dysregulation and ongoing inflammatory processes, possibly leading to increased non-AIDS-related morbidity and mortality. However, beyond the sixth month of follow-up, R5 and non-R5 patients had an almost identical risk of non-AIDS morbidity at least according to the VACS percentage risk.

The higher baseline VACS percentage risk in non-R5 patients was somewhat expected. Non-R5 patients had a higher score on at least one VACS variable (i.e. CD4 cell count); in fact, their median CD4 cell count of 168 cells/mm^3^ was significantly lower than in R5 patients; moreover it has been reported that even when the points from CD4 count are excluded, patients with a CD4 cell count <200 cells/mm^3^ have higher overall VACS scores (> 10 points) [25].

Early changes of VACS after the initiation of a successful cART in our setting resemble those from the OPTIMA study that provided an excellent demonstration of the responsiveness of the VACS index to clinical changes after treatment [30]. However, the initial reduction of VACS in our cohort was subsequently lost both in R5 and non-R5 patients; moreover, non-R5 VACS trend almost exactly fitted over the R5 trend, thereby indicating a similar increased morbidity risk in the two groups of patients. A conceivable explanation for this result could be that effective cART only influences two of the variables included in the VACS index, i.e. CD4 cell count and HIV-RNA. Therefore, the initial VACS decrease possibly reflects the prompt ART-induced immunovirological recovery, whereas the subsequent upward trend keeps the pace with changes in the remaining variables (i.e. age) included in the VACS calculation. Moreover, cART itself can negatively affect components of the VACS index either because of toxicity or because the restored sense of well-being encourages relevant changes in health risk behaviors which are prejudicial for VACS [31].

Among the limitations of our study, we need to mention the retrospective design, which prevented us to reliably collect data on non-AIDS events in our cohort.

In conclusion, our study confirms an association between pre-therapy CRT, the CD4/CD8 ratio and VACS. A successful cART regimen positively affects the CD4/CD8 ratio; however, the disadvantage conferred by a non-R5 CRT is maintained over time. The different trend of VACS percentage risk which shows a transitory decline before rising again regardless of CRT could be due to variables included in the VACS index calculation and factors adversely affecting these variables. Alternately, since VACS index has been validated to five years, a longer follow-up than in our series is necessary to perceive differences between R5 and non-R5 patients.

## Supporting information

S1 File Dataset. (XLSX)
